# FunAndes – A functional trait database of Andean plants

**DOI:** 10.1038/s41597-022-01626-6

**Published:** 2022-08-20

**Authors:** Selene Báez, Luis Cayuela, Manuel J. Macía, Esteban Álvarez-Dávila, Amira Apaza-Quevedo, Itziar Arnelas, Natalia Baca-Cortes, Guillermo Bañares de Dios, Marijn Bauters, Celina Ben Saadi, Cecilia Blundo, Marian Cabrera, Felipe Castaño, Leslie Cayola, Julia G. de Aledo, Carlos Iván Espinosa, Belén Fadrique, William Farfán-Rios, Alfredo Fuentes, Claudia Garnica-Díaz, Mailyn González, Diego González, Isabell Hensen, Ana Belén Hurtado, Oswaldo Jadán, Denis Lippok, M. Isabel Loza, Carla Maldonado, Lucio Malizia, Laura Matas-Granados, Jonathan A. Myers, Natalia Norden, Imma Oliveras Menor, Kerstin Pierick, Hirma Ramírez-Angulo, Beatriz Salgado-Negret, Matthias Schleuning, Miles Silman, María Elena Solarte-Cruz, J. Sebastián Tello, Hans Verbeeck, Emilio Vilanova, Greta Weithmann, Jürgen Homeier

**Affiliations:** 1 Departamento de Biología, Escuela Politécnica Nacional del Ecuador, Ladrón de Guevara E11-253 y Andalucía, Quito, Ecuador; 2 Biology and Geology, Physics and Inorganic Chemistry, Universidad Rey Juan Carlos, Calle Tulipán s/n, Móstoles, Madrid, Spain; 3 Departamento de Biología, Área de Botánica, Universidad Autónoma de Madrid, Madrid, Calle Darwin 2, ES–28049 Madrid, Spain; 4 Centro de Investigación en Biodiversidad y Cambio Global (CIBC-UAM), Universidad Autónoma de Madrid, Calle Darwin 2, ES–28049 Madrid, Spain; 5 Escuela de Ciencias Agrícolas, Pecuarias y del Medio Ambiente, Universidad Nacional Abierta a Distancia de Colombia, Sede José Celestino Mutis, Cl. 14 Sur 14-23, Bogotá, Colombia; 6 Instituto Experimental de Biología Luis Adam Briancon, Universidad Mayor Real y Pontificia San Francisco Xavier de Chuquisaca, Dalence 235, Sucre, Bolivia; 7 Departamento de Ciencias Biológicas y Agropecuarias, Universidad Técnica Particular de Loja, Ecuador. San Cayetano Alto s/n. Paris y Marcelino Chamagnat, 1101608 Loja, Ecuador; 8 Departamento de Biología. Grupo de Biología de Páramos y Ecosistemas Andinos, Universidad de Nariño, Calle 18 # 50-02 Ciudadela Universitaria Torobajo, Pasto, Colombia; 9 Department of Environment, CAVElab - Computational and Applied Vegetation Ecology, Ghent University, Coupure links 653, B-9000 Gent, Belgium; 10 Instituto de Ecología Regional, Universidad Nacional de Tucumán, CONICET, Residencia Universitaria Horco Molle, Edificio Las Cúpulas, 4107 Tucumán, Argentina; 11 Herbario UIS, Escuela de Biología, Universidad Industrial de Santander, Carrera. 27, calle 9a, Bucaramanga, Colombia; 12 Herbario Nacional de Bolivia, Instituto de Ecología, Universidad Mayor de San Andrés, Calle 27 s/n, La Paz, Bolivia; 13 Center for Conservation and Sustainable Development, Missouri Botanical Garden, 4344 Shaw Blvd., St. Louis, MO 63110 USA; 14 School of Geography, University of Leeds, Leeds, LS2 9JT UK; 15 Living Earth Collaborative, Washington University, 1 Brookings Drive, St. Louis, MO 63130 USA; 16 Department of Biology, University of Florida, 876 Newell Drive, ZIP 32611 Gainesville, Florida USA; 17 Instituto de Investigación de Recursos Biológicos Alexander von Humboldt, Calle 28 A # 15-09, Bogotá, Colombia; 18 Conservación Internacional, Colombia, Carrea 13 # 71-41, Bogotá, Colombia; 19 Institute of Biology/Geobotany and Botanical Garden, Martin Luther University Halle-Wittenberg, Am Kirchtor 1, D-06108 Halle, Germany; 20 Escuela de Ingeniería Agronómica, Universidad de Cuenca, Av. 12 de Abril y Av. Loja s/n, Cuenca, Ecuador; 21 Global Tree Conservation Program and the Center for Tree Science, The Morton Arboretum, Lisle, IL 60532-1293 USA; 22 Facultad de Ciencias Agrarias, Universidad Nacional de Jujuy, Alberdi 47, San Salvador de Jujuy, CP 4600 Jujuy, Argentina; 23 Department of Biology, Washington University, 1 Brookings Drive, St. Louis, MO 63130 USA; 24 AMAP (Botanique et Modélisation de l’Architecture des Plantes et des Végétations), CIRAD, CNRS, INRA, IRD, Université de Montpellier, TA-A51/PS, Boulevard de la Lironde, 34398 cedex 5 Montpellier, France; 25 Environmental Change Institute, School of Geography and the Environment, University of Oxford, South Parks Road, Oxford, UK; 26 Plant Ecology and Ecosystems Research, University of Goettingen, Untere Karspüle 2, 37073 Goettingen, Germany; 27 Instituto de Investigaciones para el Desarrollo Forestal (Indefor), Vía los Chorros de Milla, Mérida, Venezuela; 28 Departamento de Biología, Universidad Nacional de Colombia, Cra 45 #26-85, Bogotá, Colombia; 29 Senckenberg Biodiversity and Climate Research Centre (SBiK-F), Senckenberganlage 25, 60325 Frankfurt, Germany; 30 Department of Biology, Wake Forest University, Winston-Salem, NC 27109 USA; 31 Wildlife Conservation Society (WCS), 2300 Southern Boulevard Bronx, New York, 10460 USA; 32 Faculty of Resource Management, HAWK University of Applied Sciences and Arts, Büsgenweg 1 A, 37077 Goettingen, Germany; 33 Centre of Biodiversity and Sustainable Land Use (CBL), University of Goettingen, Goettingen, Germany

**Keywords:** Tropical ecology, Biodiversity, Forest ecology

## Abstract

We introduce the FunAndes database, a compilation of functional trait data for the Andean flora spanning six countries. FunAndes contains data on 24 traits across 2,694 taxa, for a total of 105,466 entries. The database features plant-morphological attributes including growth form, and leaf, stem, and wood traits measured at the species or individual level, together with geographic metadata (i.e., coordinates and elevation). FunAndes follows the field names, trait descriptions and units of measurement of the TRY database. It is currently available in open access in the FIGSHARE data repository, and will be part of TRY’s next release. Open access trait data from Andean plants will contribute to ecological research in the region, the most species rich terrestrial biodiversity hotspot.

## Abstract (summary)

**Table:** 

Measurement(s)	Bark thickness • Leaf area • Leaf aluminium (Al) content per leaf dry mass • Specific leaf area • Leaf calcium (Ca) content per leaf dry mass • Leaf carbon (C) content per leaf dry mass • Leaf carbon (C) isotope signature (delta 13 C) • Leaf compoundness • Leaf dry mass per leaf fresh mass (leaf dry matter content, LDMC) • Leaf magnesium (Mg) content per leaf dry mass • Leaf nitrogen (N) content per leaf dry mass • Leaf nitrogen (N) isotope signature (delta 15 N) • Leaf phosphorus (P) content per leaf dry mass • Leaf potassium (K) content per leaf dry mass • Leaf texture (sclerophylly, physical strength, toughness) • leaf thickness • Plant growth form • Stem conduit cross-sectional area (vessels and tracheids) • Stem conduit density (vessels and tracheids) • Sapwood specific conductivity
Technology Type(s)	bark gauge • Scanner Device • inductively-coupled plasma atomic emission spectroscopy • Calculated from area and mass • CN analyzer • Isotope analyzer • *in vivo* visual assessment • Ratio of fresh to dry mass • punch tester • micrometer • optical analysis of cross sections with specific software • weight and volume measurement • estimated with equations from wood anatomy
Factor Type(s)	Country • Lat • Long • Elevation • Collection_year
Sample Characteristic - Organism	Tracheophyta
Sample Characteristic - Environment	Andean ecosystems • cloud forest • tropical upper montane forest • tropical lower montane forest • Paramo • Andes
Sample Characteristic - Location	South America • Venezuela • Colombia • Ecuador • Peru • Bolivia • Argentina

## Background & Summary

Functional traits are measurable properties of a plant describing its structure, function or life history strategy that determine species responses to biotic and abiotic environmental conditions across scales of biological complexity, from communities to ecosystems^1–4^. Exploring variation in plant functional traits provides key insights into plant species distribution, community assembly mechanisms, evolutionary strategies, and ecosystem level potential responses to global environmental change^5–13^. Global databases of plant functional traits currently feature an unprecedented amount of trait information that supports scientific work on plant functional ecology, including BIEN^14^, GIFT^15^, and TRY^16,17^. Yet, the geographical coverage of trait measurements still remains limited for highly diverse tropical areas, especially in mountainous regions^15,16^.

**Fig. 1:**
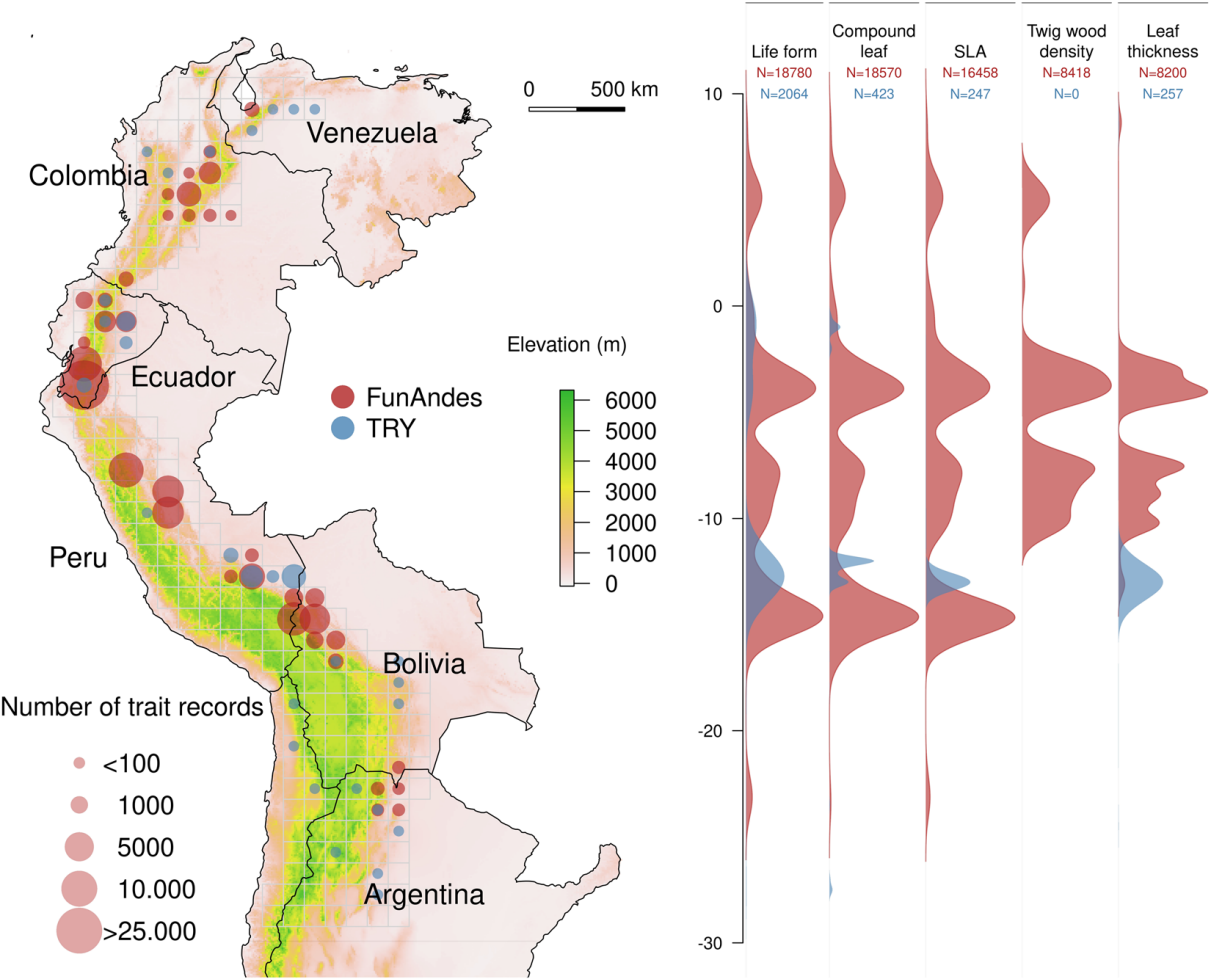
Geographic distribution of plant traits in FunAndes and TRY version 5^17^ in 1-degree cells (~1 km). Montane sites above 500 m of elevation and buffer areas of 50 km below such elevation show density distribution of the most representative plant traits in FunAndes and TRY along the latitudinal gradient.

The tropical Andes is a major hotspot of global biodiversity and endemism. With about 2% of the terrestrial area of the planet, it holds 10% of the species of vascular plants^18^^–^^20^. However, trait information for Andean plants is underrepresented in global plant trait databases. These information gap limits our understanding of variation in plant trait composition and diversity at regional, continental, and global scales. Synthesizing and harmonizing trait measurements from remote and understudied areas is critical for global and regional data archiving initiatives^21^, and for advancing empirical biodiversity research. Here, we present the FunAndes database, a compilation of plant functional traits in the tropical Andes (Fig. 1). The records in FunAndes stem from 18 unpublished datasets contributed by different research groups conducting fieldwork in the region. FunAndes follows the structure and terminology of the TRY database, and is available in the FIGSHARE data repository^22^. In total, FunAndes contains 105,466 records of 24 traits, covering 2,694 Andean (morpho-) species in 670 genera and 175 families. Assembling FunAndes encompassed the following steps: 1) developing a TRY-based format for data contributors, 2) revising comparability among protocols used for trait data collection, 3) checking trait measurement units for each contributed dataset, 4) detecting and deleting suspicious or erroneous trait measurements, 5) compiling the contributed data into a unique source with common taxonomic names, units, and terminology. To our knowledge, FunAndes is the first open access trait database of the Andean flora, filling a substantial gap in global functional trait data. We hope that providing a standardized and curated database on Andean plant traits will encourage plant trait ecological research in Andean ecosystems, as well as comparative studies across tropical regions.

## Methods

### Primary sources

We first developed a basic data template containing trait names, trait descriptions and units of measurement, together with information (e.g., site coordinates and collection dates, number of samples collected). This template was distributed to potential data contributors, scientists collecting vascular plant functional trait data mainly in tropical forests of the Andean region. Filled templates were returned to the writing team, and FunAndes was assembled from 18 distinct datasets containing field data of Andean plant traits (Tables 1 and 2).

**Table 1:** Species and trait observations per country in FunAndes.

	Country	SpeciesNames (n)	Trait observations (n)	Trait observations (%)
1	Argentina	97	1457	1.38
2	Bolivia	692	19,463	18.45
3	Colombia	294	7,150	6.78
4	Ecuador	1170	50,401	47.79
5	Peru	1287	26,372	25.01
6	Venezuela	27	623	0.59
	Total	NA	105,466	100

**Table 2:** Summary of the 18 datasets inFunAndes.

Dataset ID	PI LastName	PI FirstName	Country	Number of entries
ABERG	Farfán-Ríos	William	Peru	2522
Amira Project	Apaza	Amira	Bolivia	2040
BAMBOOTRAITS	Fadrique	Belen	Peru	1860
BOTROPANDES ECU	Bañares de Dios	Guillermo	Ecuador	9083
BOTROPANDES	Bañares de Dios	Guillermo	Peru	9225
COFOREC	Bauters	Marijn	Ecuador	996
DISPLAMAZ	Macía	Manuel J.	Peru	12907
E., ALVAREZ TRAIT DATABASE	Alvarez-Davila	Esteban	Colombia	623
FPY	Blundo	Cecilia	Argentina	1457
Homeier Projects	Homeier	Jürgen	Bolivia	1015
Homeier Projects ECU	Homeier	Jürgen	Ecuador	30557
Iguaque	Salgado-Negret	Beatriz	Colombia	2272
Jadan Project	Jadán	Oswaldo	Ecuador	9623
LCP UDENAR IAVH	Solarte	Maria Elena	Colombia	611
Madidi Project	Tello	J Sebastian	Bolivia	16408
Rastrojos	Norden	Natalia	Colombia	3008
Sumapaz-Cruz Verde	Garnica-Díaz	Claudia	Colombia	636
VEN-SEU	Vilanova	Emilio	Venezuela	623
Total				105,466

### Trait definitions and protocols

Trait definitions and trait units of measurement in FunAndes follow those of the TRY database, for a total of 24 plant traits, two categorical and 22 numerical (Table 3). All trait data contributed to FunAndes were obtained from individuals growing in natural vegetation, following standard and comparable methods^23,24^. Furthermore, traits were measured mostly in adult individuals, never in seedlings or saplings. Leaf traits were quantified from exposed mature leaves in the plant canopy. A summary of trait geographical representation in FunAndes is presented in Fig. 1. A comparison between trait data in FunAndes and TRY version 5^17^ is presented in Table 4.

**Table 3:** Plant functional traits represented in FunAndes. Trait definitions and units of measurement follow those of TRY^16^ (https://www.try-db.org/de/TabDetails.php).

Trait Name	Unit
Bark thickness	mm
Leaf area (in case of compound leaves: leaf, petiole excluded)	mm^2^
Leaf area (in case of compound leaves: leaf, petiole included)	mm^2^
Leaf aluminium (Al) content per leaf dry mass	mg g^−1^
Leaf area per leaf dry mass (specific leaf area, SLA or 1/LMA): petiole included	mm^2^ mg^−1^
Leaf area per leaf dry mass (specific leaf area, SLA or 1/LMA) petiole, rhachis and midrib excluded	mm^2^ mg^−1^
Leaf calcium (Ca) content per leaf dry mass	mg g^−1^
Leaf carbon (C) content per leaf dry mass	mg g^−1^
Leaf carbon (C) isotope signature (delta 13 C)	mg kg^−1^
Leaf compoundness	unitless
Leaf dry mass per leaf fresh mass (leaf dry matter content, LDMC)	mg g^−1^
Leaf magnesium (Mg) content per leaf dry mass	mg g^−1^
Leaf nitrogen (N) content per leaf dry mass	mg g^−1^
Leaf nitrogen (N) isotope signature (delta 15 N)	mg kg^−1^
Leaf phosphorus (P) content per leaf dry mass	mg g^−1^
Leaf potassium (K) content per leaf dry mass	mg g^−1^
Leaf texture (sclerophylly, physical strength, toughness)	kN m^−1^
Leaf thickness	mm
Plant growth form	unitless
Stem conduit cross-sectional area (vessels and tracheids)	μm
Stem conduit density (vessels and tracheids)	mm^−2^
Stem dry mass per stem fresh volume (stem specific density, SSD, wood density): branch	g/cm^3^
Stem dry mass per stem fresh volume (stem specific density, SSD, wood density): sapwood	g/cm^3^
Wood (sapwood) specific conductivity (stem specific conductivity)	kg m^−1^ Mpa^−1^ s^−1^

**Table 4:** Plant functional traits inFunAndes in comparison to TRY version 5^17^ for the Andean region.

Trait	FunAndes				TRY
	Number of Project IDs	Entries	Entries identified to genus or species level	Species	Entries
Bark thickness	2	1242	1232	340	0
Leaf aluminium (Al) content per leaf dry mass	2	1712	1689	402	318
Leaf area (in case of compound leaves: leaf, petiole excluded)	2	686	670	162	0
Leaf area (in case of compound leaves:leaf, petiole included)	10	6512	6399	1534	0
Leaf area per leaf dry mass (specific leaf area, SLA or 1/LMA) petiole, rhachis and midrib excluded	2	681	665	161	247
Leaf area per leaf dry mass (specific leaf area, SLA or 1/LMA): petiole included	14	16458	15577	2423	0
Leaf calcium (Ca) content per leaf dry mass	3	2289	2213	558	318
Leaf carbon (C) content per leaf dry mass	5	2785	2700	654	882
Leaf carbon (C) isotope signature (delta 13 C)	2	259	257	72	68
Leaf compoundness	18	18570	17642	2600	423
Leaf dry mass per leaf fresh mass (leaf dry matter content, LDMC)	6	2058	2049	403	68
Leaf magnesium (Mg) content per leaf dry mass	2	2096	2023	519	318
Leaf nitrogen (N) content per leaf dry mass	6	2853	2768	669	1698
Leaf nitrogen (N) isotope signature (delta 15 N)	2	259	257	72	0
Leaf phosphorus (P) content per leaf dry mass	4	2378	2302	577	1566
Leaf potassium (K) content per leaf dry mass	2	2170	2096	523	318
Leaf texture (sclerophylly, physical strength, toughness)	2	1423	1407	345	0
Leaf thickness	7	8200	7414	1779	257
Plant growth form	17	18780	17818	2657	2064
Stem conduit cross-sectional area (vessels and tracheids)	1	933	912	367	3
Stem conduit density (vessels and tracheids)	1	930	909	367	0
Stem dry mass per stem fresh volume (stem specific density, SSD, wood density) branch	8	8418	7625	1795	0
Stem dry mass per stem fresh volume (stem specific density, SSD, wood density) sapwood	5	2845	2814	683	0
Wood (sapwood) specific conductivity (stem specific conductivity)	1	929	908	367	3
**Total**		105,466	100,346	20,029	**8551**

### Database structure

The database contains 24 fields to provide contextual information about data collection, including association of trait data to permanent vegetation plots, site coordinates and collection dates; and information about the trait value provided (e.g., if the value provided is a single observation or an average of trait measurements) (Table 5).

**Table 5:** Definitions of fields in the FunAndes database.

Number	Field	Definition
1	Project_ID	Project name of the contributed dataset
2	Plot_ID	Plot identification code
3	Plant_ID	Plant identification code or voucher
4	Sample_ID	Sample number
5	SpeciesOriginal	Species of the plant in the original dataset
6	OrderLCVP	Taxonomic order provided by the Leipzig Cataloge of Vascular Plants
7	FamilyLCVP	Taxonomic family provided by the Leipzig Cataloge of Vascular Plants
8	GenusLCVP	Taxonomic genus provided by the Leipzig Cataloge of Vascular Plants
9	SpeciesLCVP	Taxonomic species provided by the Leipzig Cataloge of Vascular Plants
10	Long	Longitude in decimal degrees
11	Lat	Latitude in decimal degrees
12	Elevation	Elevation in m
13	Country	Country
14	Collection_year	Year of collection
15	ValueKindName	Value kind (single measurement, mean, median, etc.)
16	SpeciesName	Revised species name
17	OrigValueStr	Trait value
18	OriginalName	Trait name following TRY
19	OrigUnitStr	Trait units
20	LastName	Last Name of the PI contributing the dataset
21	FirstName	First Name of the PI contributing the dataset
22	Email	Email of the PI of the contributed dataset
23	Dataset	Identifier of the dataset in TRY (FunAndes)
24	Observation_ID	Unique identifier of each observation in FunAndes

### Harmonization

We followed various steps to ensure the quality of the data before adding a contributed dataset to FunAndes. Our workflow consisted of a series of operations, including generating dataset IDs for each contributed dataset, harmonizing data into common measurement units, translating terms (trait values) for categorical variables, verifying and correcting collection coordinates, and identifying erroneous trait data measurements. Each data contributor was contacted to double check methods used for trait collection, correct or eliminate suspicious trait values. Finally, duplicates were removed to create the final version of the database. All steps taken toward data standardization were done in R^16^ using built-in functions and the package ‘dplyr’^25^.

### Taxonomy

Species names standardization was conducted with the R package ‘LCVP’ of The Leipzig Catalogue of Vascular Plants^18^. Original species names were compared to LCVP names by searching for matches. Non-matches (mainly caused by incorrect spelling) were revised by an expert in Andean flora (J.H.), and corrected following LCVP. The final FunAndes database reports both the original and the updated taxon name alongside each trait record. For each morphospecies, higher taxonomic affiliations obtained from the LCVP were included.

## Data Records

### Access

FunAndes database is stored and available for direct download from the FIGSHARE data repository^22^ and will become available from the TRY Plant Trait Database in the next release (https://www.try-db.org).

### Data coverage

FunAndes includes 105,466 trait records for 24 traits of 2,694 Andean morpho-species in 670 genera and 175 taxonomic families. Therefore, FunAndes presents trait information for roughly nine percent of the ~30,000 species of vascular plants estimated to occur in the Tropical Andes^20,26^. Three traits of FunAndes (plant growth form, leaf compoundness, specific leaf area) make up half of the records in the database (Table 4). Leaf trait data make up 67.7% of the database, followed by whole plant (i.e., plant growth form and leaf compoundness) (17.8 and 17.6%, respectively) and stem traits (14.5%). Each species has an average of 7.4 (SD = 5.1) distinct traits. All observations have geographic coordinates.

**Fig. 2:**
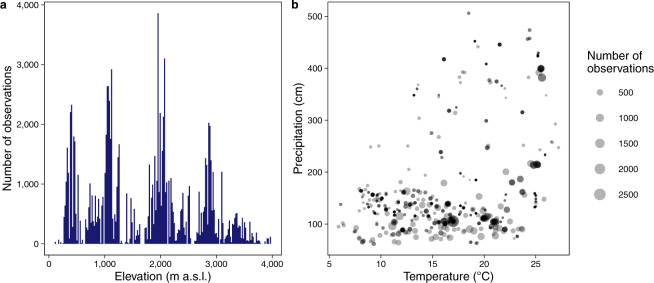
Distribution of plant trait data in FunAndes along gradients of (**a**) elevation, (**b**) Mean annual temperature and Mean total annual precipitation. Climatic variables were extracted from the Chelsa climate database^27^.

Considering the Andean countries, Ecuador has 47.8% of all the trait observations in FunAndes, followed by Peru (25.0%) and Bolivia (19.5%) (Fig. 1, Table 1). Data in FunAndes comes from 788 collection sites (i.e., unique combinations of latitude and longitude) and is associated to 570 forest plots. Furthermore, trait observations are grouped mainly around 500, 1,000, 2,000 and 3,000 m of elevation (Fig. 2a). The data is widely distributed along a gradient of mean annual temperature, but clustered toward lower values of total mean annual precipitation (Fig. 2b).

**Fig. 3:**
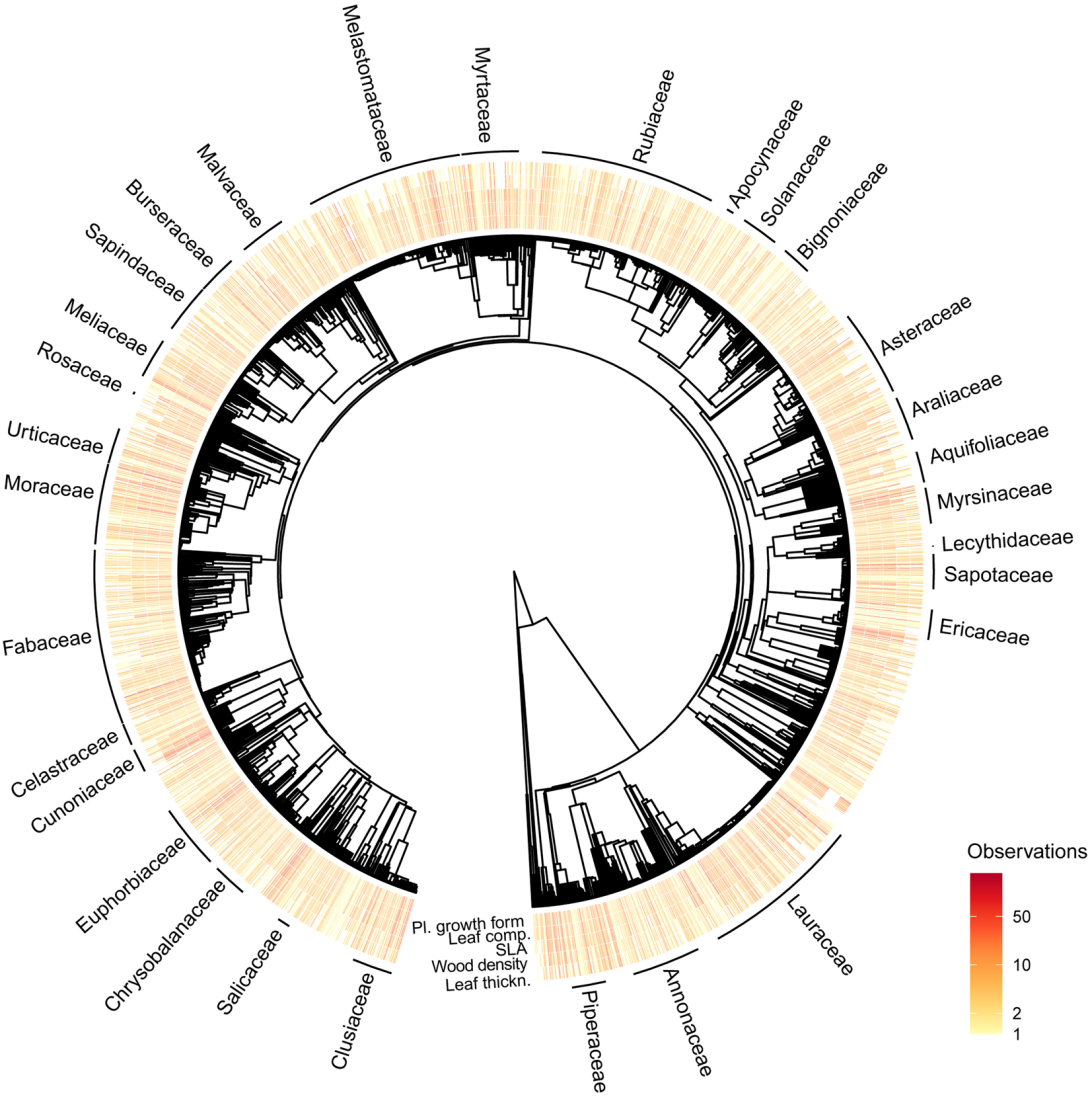
Phylogenetic distribution of trait data in FunAndes showing the total number of observations per taxa for the five most represented functional traits: Plant growth form, leaf compoundness, specific leaf area (SLA), wood density, and leaf thickness. The phylogenetic tree shows information for 150 families and 2,690 species. The tree is based on a recent plant phylogeny^28^, nomenclature of The Plant List (http://www.theplantlist.org), and was created with the package ‘V.phylomaker’ ^29^.

The five most represented plant functional traits in FunAndes - plant growth form, leaf compoundness, specific leaf area (SLA), wood density, leaf thickness - are homogeneously distributed in the tree phylogeny (Fig. 3).

TRY version 5^23^ hosts 8,548 entries for Andean plants, corresponding to 1,123 species, and 15 of the 24 functional traits held in FunAndes (Table 4). FunAndes, therefore, will increase available trait data by a factor of 12, and at least double the current representation of traits per species in TRY. In consequence, FunAndes is a substantial contribution to plant functional trait data availability for the Andean region.

## Technical Validation

For each contributed dataset we visually inspected all data and metadata producing histograms of each trait value to identify outliers or mistaken measures. In most cases, extreme values were discussed with data contributors to make decisions toward correcting or eliminating erroneous observations. With the final version of the database, histograms were produced once again to check for outliers or mistaken values.

## Usage Notes

The data can be downloaded from the FIGSHARE data repository under the terms of Creative Commons Zero (CC0) waiver. We also provide FunAndes database in the TRY Plant Trait Database (https://www.try-db.org). Users of FunAndes data are invited to cite this publication: Báez *et al*. xx. FunAndes – A functional trait database of Andean plants. Scientific Data. 00:00-00, and the accompanying FIGSHARE dataset^22^.

## Data Availability

The contributed datasets were provided in Excel spreadsheets (Microsoft Office 2013), therefore no code is available for this step. Scripts to conduct taxonomic standardization using the LCVP, to plot environmental distribution, and trait representation in the plant phylogeny are available at FIGSHARE^22^. The scripts were developed in R.

## References

[1] Violle C (2007). Let the concept of trait be functional!. Oikos.

[2] Enquist, B. J. *et al*. In *Advances in Ecological Research* Vol. Volume 52 (eds G., Woodward, S., Pawar & Dell Anthony, I.) 249–318 (Academic Press, 2015).

[3] Suding KN (2008). Scaling environmental change through the community-level: a trait-based response-and-effect framework for plants. Global Change Biology.

[4] Reich PB (2014). The world-wide ‘fast–slow’ plant economics spectrum: a traits manifesto. Journal of Ecology.

[5] Báez S, Fadrique B, Feeley K, Homeier J (2022). Changes in tree functional composition across topographic gradients and through time in a tropical montane forest. PLOS ONE.

[6] Umaña MN, Zhang C, Cao M, Lin L, Swenson NG (2017). A core-transient framework for trait-based community ecology: an example from a tropical tree seedling community. Ecology Letters.

[7] Báez S, Homeier J (2018). Functional traits determine tree growth and ecosystem productivity of a tropical montane forest: Insights from a long‐term nutrient manipulation experiment. Global Change Biology.

[8] Sanchez-Martinez P, Martínez-Vilalta J, Dexter KG, Segovia RA, Mencuccini M (2020). Adaptation and coordinated evolution of plant hydraulic traits. Ecology Letters.

[9] Wieczynski DJ (2019). Climate shapes and shifts functional biodiversity in forests worldwide. Proceedings of the National Academy of Sciences.

[10] Bjorkman AD (2018). Plant functional trait change across a warming tundra biome. Nature.

[11] Wright IJ (2017). Global climatic drivers of leaf size. Science.

[12] Bañares-de-Dios G (2020). Linking patterns and processes of tree community assembly across spatial scales in tropical montane forests. Ecology.

[13] Homeier J, Seeler T, Pierick K, Leuschner C (2021). Leaf trait variation in species-rich tropical Andean forests. Scientific Reports.

[14] Enquist, B. J., Condit, R., Peet, R. K., Schildhauer, M. & Thiers, B. *The botanical information and ecology network (BIEN): cyberinfrastructure for an integrated botanical information network to investigate the ecological impacts of global climate change on plant biodiversity*, www.iplantcollaborative.org/sites/default/files/BIEN_White_Paper.pdf (2009).

[15] Weigelt P, König C, Kreft H (2020). GIFT – A Global Inventory of Floras and Traits for macroecology and biogeography. Journal of Biogeography.

[16] Kattge J (2011). TRY – a global database of plant traits. Global Change Biology.

[17] Kattge J (2020). TRY plant trait database – enhanced coverage and open access. Global Change Biology.

[18] Rahbek C (2019). Humboldt’s enigma: What causes global patterns of mountain biodiversity?. Science.

[19] Antonelli A (2018). Geological and climatic influences on mountain biodiversity. Nature Geoscience.

[20] Mittermeier, R. A., Turner, W. R., Larsen, F. W., Brooks, T. M. & Gascon, C. in *Global biodiversity conservation: the critical role of hotspots* (ed and Habel, J. C., Zachos, F. E.) 3–22 (Heidelberg: Springer–Verlag Berlin, 2011).

[21] Mariano E (2021). LT-Brazil: A database of leaf traits across biomes and vegetation types in Brazil. Global Ecology and Biogeography.

[22] Báez S (2022). FIGSHARE.

[23] Pérez-Harguindeguy N (2016). New handbook for standardised measurement of plant functional traits worldwide. Australian Journal of Botany.

[24] Cornelissen JHC (2003). A handbook of protocols for standardised and easy measurement of plant functional traits worldwide. Australian Journal of Botany.

[25] dplyr: A Grammar of Data Manipulation v. R package version 1.0.7 (2021).

[26] Pérez-Escobar OA (2022). The Andes through time: evolution and distribution of Andean floras. Trends in Plant Science.

[27] Karger DN (2017). Climatologies at high resolution for the earth’s land surface areas. Scientific Data.

[28] Smith SA, Brown JW (2018). Constructing a broadly inclusive seed plant phylogeny. American Journal of Botany.

[29] Jin Y, Qian H (2019). V.PhyloMaker: an R package that can generate very large phylogenies for vascular plants. Ecography.

